# Neuroprotection and Neuroplasticity Through Natural Bioactive Compounds: An Editorial Overview

**DOI:** 10.3390/ijms27041788

**Published:** 2026-02-13

**Authors:** Terezia Kiskova-Simkova

**Affiliations:** Institute of Pathology, Faculty of Medicine, Pavol Jozef Safarik University in Kosice, Rastislavova 43, 040 01 Kosice, Slovakia; terezia.simkoval@upjs.sk

Brain diseases are the leading cause of disability-adjusted life years (DALYs) and the second-leading cause of death [1,2]. As the brain is a vulnerable structure, it may be damaged during development as well as during adulthood. This is why the scientific and lay communities are focused on exploring how various bioactive compounds may promote neuroprotection and prevent/treat brain diseases, such as neurodegenerative disorders, ischemic attacks, brain cancer, or depression.

Natural products and their structural analogs have historically made a major contribution to pharmacotherapy [3,4]. Natural products are organic compounds produced by living organisms: predominantly plants, microorganisms, or marine species but also animals [5,6]. McDougall defined traditional medicine as follows: “The term traditional medicine encompasses both codified and non-codified health-care systems, including practices, skills, knowledge, and philosophies rooted in diverse historical and cultural contexts, that are distinct from, and predate, modern biotechnology. This vast and varied body of knowledge, including knowledge about the medical products used, represents a rich resource, but tapping into it can be challenging” [7]. Traditional medicine remains a globally utilized healthcare resource, firmly grounded in cultural heritage, historical experience, and local practices. The WHO Global Traditional Medicine Strategy 2025–2034 aims to enhance universal health coverage through the systematic integration of evidence-based traditional, complementary, and integrative medicine within national health systems [8]. Food-medicine homology (FMH) is a traditional concept where certain natural materials act as both food and medicine, offering both nutritional value and therapeutic benefits. FMH is evolving from an experience-driven discipline into a scientifically grounded field supported by modern methodologies. From 2026 onward, this transformation will rest on four pillars: establishing trust through rigorous science, advancing mechanistic insight via systematic analysis, enhancing health outcomes through precision medicine, and upgrading the industrial ecosystem through cutting-edge technologies [9].

There are several classes of bioactive compounds within the brain that could be stimulated via natural compounds [10,11]. Natural compounds can influence the levels and activity of neurotransmitters in the brain, which are essential for proper neuronal communication [12]. Certain phytonutrients influence the production and regulation of neurotransmitters, such as serotonin and dopamine, which are key chemicals involved in mood regulation and emotional well-being [12]. Other bioactive molecules involved in the brain are neurotrophic factors and growth regulators, such as brain-derived neurotrophic factor (BDNF) [13]; hormones and neurosteroids, such as vitamin D [14]; and lipid mediators and metabolic signaling molecules. While the benefits of a nutrient-dense diet for physical health are well established, recent technological and imaging advances increasingly highlight its important role in brain health as well [10].

Within the Special Issue titled “Bioactive Compounds in Human Brain Structures and Diseases: 2nd Edition”, five interesting research articles have been published. Interestingly, three of them focus on depression. Depression has currently become one of the leading disabilities, so it is critical to ensure proper management through personalized treatment and psychiatric interventions, leading to lower treatment resistance and higher compliance.

Natural compounds have been shown to possess the ability to influence depression in many ways, including effects on brain structures, such as the hippocampus. It has been shown that hippocampal neurogenesis is suppressed during depression [15]. In the special issue, several natural compounds have been studied, such as resveratrol, showing antidepressant effects, accompanied by the restoration of impaired hippocampal neuroplasticity, or an extract from *Withania somnifera*, known as ashwagandha, showing improvement in cell loss, during restraint stress-induced anxiety-like behavior. In the special issue, also a lichen secondary metabolite, gyrophoric acid, reported that the changes in behavior may reflect the potential antidepressant effects of this molecule. Indeed, gyrophoric acid has been later shown to influence not only behavior but also to improve hippocampal neurogenesis during stress-induced depression [16].

The most commonly prescribed antidepressants are drugs that modulate neurotransmitters, including selective serotonin reuptake inhibitors, serotonin–norepinephrine reuptake inhibitors, and tricyclic and monoamine oxidase inhibitors. Despite their widespread use, these treatments show only moderate efficacy and can cause significant side effects, especially during chronic administration. Consequently, considerable research efforts have focused on identifying alternative therapeutic approaches, especially those involving natural products [17].

## Data Availability

Not applicable.
